# Effect of Ball-Milling on Starch Crystalline Structure, Gelatinization Temperature, and Rheological Properties: Towards Enhanced Utilization in Thermosensitive Systems

**DOI:** 10.3390/foods12152924

**Published:** 2023-07-31

**Authors:** Matheus de Oliveira Barros, Adriano Lincoln Albuquerque Mattos, Jessica Silva de Almeida, Morsyleide de Freitas Rosa, Edy Sousa de Brito

**Affiliations:** 1 Department of Chemical Engineering, Federal University of Ceará (UFC), Fortaleza 60455-760, Brazil; matheus.oliveira_@hotmail.com.br (M.d.O.B.); jeh.quimica@gmail.com (J.S.d.A.); 2 Embrapa Tropical Agroindustry, Rua Dra Sara Mesquita 2270, Fortaleza 60511-110, Brazil; adriano.mattos@embrapa.br (A.L.A.M.); morsyleide.rosa@embrapa.br (M.d.F.R.); 3 Embrapa Food and Territories, Rua Cincinato Pinto 348, Maceió 57020-050, Brazil

**Keywords:** ball milling, corn starch, gelatinization temperature

## Abstract

Starch’s crystalline structure and gelatinization temperature might facilitate or hinder its use. Ball milling has frequently been mentioned in the literature as a method for reducing starch size and as a more environmentally friendly way to change starch, such as by increasing surface area and reactivity, which has an impact on other starch properties. In this study, starch samples were milled for varying durations (1, 5, 10, 20, and 30 h) and at different starch-to-ball mass ratios (1:6 and 1:20). Microscopy and XRD revealed that prolonged milling resulted in effective fragmentation and a decrease in crystallinity of the starch granules. Increasing milling times resulted in an increase in amylose content. Rheology and thermal studies revealed that gelatinization temperatures dropped with milling duration and that viscosity and thixotropy were directly influenced. The samples milled for 10, 20, and 30 h at a ratio of 1:20 were the most fragmented and upon drying formed a transparent film at ambient temperature, because of the lower gelatinization temperature. Starch ball milling could lead to the use of this material in thermosensitive systems.

## 1. Introduction

Starch, a biopolymer and one of the main polysaccharides on the planet, is a natural and abundant molecule extracted from various plants, such as but not limited to cassava, corn, and potatoes [1]. Anhydroglucose forms a linear chain structure linked by glycosidic linkages (α 1-4). These linear chains link with each other, forming amylose (AM) and amylopectin (AP). Together, AM and AP form alternating crystalline and amorphous lamellae, which are arranged into growth rings originating from the hilum, forming the starch granule [2,3].

Starch’s main components (amylose and amylopectin) have entirely different properties. Amylose, a macromolecule formed by glucose units joined by α-1,4 glycosidic bonds, gives rise to a primarily linear chain with a few long ramifications. Amylopectin, formed by glucose units joined by α-1,4 and α-1,6 glycosidic bonds, creates a smaller, highly branched structure that is organized in a double helix conformation. As a result, amylose tends to retrograde, producing tough gels and strong films, while amylopectin dispersed in water forms soft gels and fragile films [4]. Due to its structured chains, amylopectin is more influential in the crystallinity of the starch granule than amylose, which is usually related to the amorphous region of the granule. A higher amylose content is associated with lower plasticization and gelatinization temperatures. The proportions in which these structures appear in starch vary according to the source [5,6]. These two components create a semicrystalline structure that results in different crystallinities and assorted sizes of starch granules, ranging from 0.5 to 175 μm, depending on the starch source [1,7].

The scope of starch application in its natural form is enhanced and limited due to its unique structure. For example, when in water and subjected to heating (60–90 °C), it forms a viscous paste that becomes a brittle gel after cooling, which can be desirable or not, depending on the final application. Another example of its unique structure influencing the application is its semicrystalline organization. The compact molecules in the crystalline region inhibit water and other chemicals from accessing and reacting with the molecules in this region, increasing the gelatinization temperature and decreasing the starch chemical reactivity [8]. Several studies have modified starch using chemicals (hydrolysis, oxidation, etc.) and enzymes to improve properties such as reactivity and surface area and enable the application of this material in different sectors, such as suspension stabilizers, thermoplastic starch, packaging, etc. [9,10,11].

A greener route to modify starch would be the milling process, as it is a process that generates no residues that could harm the environment, unlike hydrolysis, for example, which uses acids and/or organic solvents. Starch milling is widely reported in the literature as a technique for reducing the size of starch granules and ensures higher safety, simplicity, and cost-efficiency compared to other treatments. Ball milling performs the partial breakdown of the starch structure through friction, impact, and shear, caused by the collision of the balls with the starch and the starch with the mill wall, increasing the surface area and reactivity and reducing the granule size of the starch [12]. However, this reduction in the size of the granules and partial breakdown of the crystalline structure of the starch may reflect a change in other properties, such as the level of chain organization, crystallinity, and amylose content. Additionally, ball milling is reported to directly affect the starch granules’ surface area [7,13,14].

As a result, this research aims to study the thermal and rheological characteristics of these starches by using ball milling processing, which affects the granule surface area, as a greener way to alter starch properties, particularly its gelatinization temperature, and determine the structural changes affected by milling time (1, 5, 10, 20, and 30 h). Further considerations on possible applications for the obtained starches are discussed.

## 2. Materials and Methods

### 2.1. Corn Starch

This work used commercially available food-grade corn starch (Kimimo, Brazil) as feedstock, with a humidity content of 11%.

### 2.2. Starch Ball Milling

A ceramic ball mill (20 cm diameter) with ceramic balls (1.5 cm) was used with two different starch/ball mass ratios, 1:6 and 1:20 [8,12]. Operation was performed at room temperature (30 °C) and 230 rpm for 1 h, 5 h, 10 h, 20 h, and 30 h.

### 2.3. Microscopy Analysis

For scanning electron microscopy (SEM), starch samples were mounted in stubs, covered with a thin layer of gold in a metallic covering device (Emitech, model K550, Quorum Technologies, Lewes, UK), and visualized under a voltage acceleration of 15 kV in different increments on a Quanta 450-FEG, FEI.

Light microscopy analysis was performed to observe the Maltese cross in the starch granules using an optical microscope with polarized light. Samples of starch (0.01 g) were added to 1 mL of a glycerol solution (50% m/v), and 0.5 mL of this suspension was transferred to a microscope slide and covered with a coverslip for visualization under polarized light.

### 2.4. Fourier Transform Infrared Spectroscopy (FTIR)

The spectra of the starch samples were obtained using a PerkinElmer^®^ Spectrum Two spectrometer (PerkinElmer, Houston, TX, USA) with a resolution of 4 cm^−1^, wavelength range from 4000 to 600 cm^−1^, and 32 scans. The starch spectra were obtained via the attenuated total reflection (ATR) method.

The effect of ball milling on the ordered structure of the starch was also evaluated using FTIR spectra. It has been reported that bands between 950 and 1100 cm^−1^ are sensitive to changes in the starch structure, especially those at approximately 995 and 1044 cm^−1^, where the first is commonly related to the amorphous structure of the starch and the latter to the ordered structure of the starch [15,16]. Therefore, the 995:1044 cm^−1^ intensity ratio was used to indicate the degree of order in the starch structure.

### 2.5. Amylose Content

The amylose content was determined based on the methodology proposed by Chrastil et al. and Hu et al. [17,18], with modifications. In test tubes, 0.1 g of starch, 1.0 mL of absolute ethanol, and 10 mL of a solution of sodium hydroxide (1 M) were added. The tubes were heated in a water bath (70 °C, 15 min), shaken at 5 min intervals, and then cooled to room temperature in a cold-water bath. In a 50 mL beaker, 0.5 mL of the solubilized starch, 2.0 mL of iodine solution (I2 + KI; 0.01 M), 1.0 mL of acetic acid solution (1 M), and 46 mL of distilled water were added. This solution reacted at room temperature for 10 min. The absorbance reading was performed in a spectrophotometer (Shimadzu, Kyoto, Japan) at 620 nm using an acrylic cuvette with 1.4 mL of a starch solution with iodine and acetic acid and 1.4 mL of distilled water, and the absorbance was compared with the calibration curve. The blank reading was carried out using only distilled water.

### 2.6. Thermogravimetric Analysis (TGA)

Thermogravimetric measurements were performed using starch samples (~10 mg) in a PerkinElmer^®^ TGA 6000 instrument (PerkinElmer, Inc., Waltham, PA, USA) with temperatures ranging from 50 to 600 °C, a heating rate of 10 °C/min, under a nitrogen atmosphere with a flow of 40 mL/min.

### 2.7. Differential Scanning Calorimetry (DSC)

DSC analysis was performed in a TA Instruments DSC (model Q20, TA Instruments, New Castle, DE, USA) with a hermetic aluminum pan. The pans were hermetically sealed after being filled with approximately 1.5 mg of the starch sample and 5 µL of distilled water. The temperature ranged from 30 to 100 °C, with a heating ramp of 10 °C/min, and the experiment was carried out in a nitrogen atmosphere.

### 2.8. X-ray Diffraction (XRD)

For the polycrystalline samples, the DRX diffraction graphs were produced using a Co tube at 40 kV and 40 mA with a diffractometer, model XpertPro MPD—Panalytical (Panalytical, Eindhoven, The Netherlands). The sweep speed was 0.5 °/min, and the 2-theta angle ranged from 5 to 40°. Diffractogram deconvolution was performed, and the crystallinity index (CrI), which measures the proportion of crystalline peak regions to all deconvoluted peaks, was determined using the equation below Equation (1).

(1) $$CrI\;\left(\%\right)=\left(crystalline\;region\;area/total\;area\right)$$

### 2.9. Swelling Power (SP) and Solubility (S%)

*SP* and *S*% were calculated using a modified version of the approach described by Fu et al. and Mandala et al. [19,20]. A centrifuge tube with a screw lid was filled with 0.4 g (*W*_0_) of the starch samples. After adding distilled water to achieve a final starch concentration of 2.0% (w/w), the tubes were vortexed at room temperature (25 °C). Finally, the tubes were centrifuged for 15 min at 1006 g. The supernatant was carefully drained off in a petri dish, and the residue weight was noted (*W_p_*). The tubes with the residue and the petri dishes with the supernatant were dried in an air oven (105 °C) until constant weight (*W_d_* and *W_s_*, respectively). The swelling power (*SP*) and the solubility (*S*%) of the samples were calculated using the equations below Equations (2) and (3):

(2) $$SP=W_{p}/W_{d}$$

(3) $$S\%=\left(W_{s}/W_{0}\right)\times 100$$

### 2.10. Rheology

Rheological studies were conducted in the linear viscoelastic zone of the samples using a Thermo Scientific Rheometer (HAAKE MARS, Thermo Fisher Scientific, Waltham, MA, USA). The samples for gelatinization temperature were prepared by making a 5% (w/w) starch suspension and using a gelatinized starch solution (5% starch in water heated at 90 °C for 10 min), as a 5% gelatinized starch solution is more viscous than water, the starch solution was employed in place of water to reduce the effects of starch precipitation. The samples for viscosity (oscillatory rheology experiment) and thixotropy analysis were prepared by heating to 90 °C for 10 min in a 5% suspension of the starch samples in distilled water.

An oscillatory rheology experiment was performed for 2 min at 25 °C and 1% deformation using a cone plate geometry with a gap of 0.05 mm (C60/1-TiL) to estimate the dynamic viscosity, storage module (G′), and loss module (G″) of the samples. The same measuring geometry was used for gelatinization temperature measurements in a rotational experiment with a 1 s^−1^ sheer rate and a heating rate of 7.2 °C/min from 30 to 75 °C. The gelatinization temperature was defined as the temperature at which the sample began to undergo a sudden change in viscosity.

The thixotropy measurements were performed in a rotational experiment utilizing the previously described geometry. The shear rate was increased linearly from 0.1 to 10 s^−1^ in 120 s and then held constant at 10 s^−1^ for 30 s before decreasing (linearly) to 0.1 s^−1^ in 120 s. The areas of thixotropy (defined as work performed for the demolition and reconstruction of a sample inner structure) were computed by adding the areas of certain trapeziums between the “up” and “down” curves.

### 2.11. Film-Forming Ability

For the film-forming ability assays, milled starch aqueous suspensions (10%, w/w) were prepared at room temperature, subsequently poured into Petri dishes, and then dried for 16 h in a vacuum oven at 30 °C. The resulting films were registered with pictures to observe the effect of milling on the starch film forming capacity at low temperatures.

### 2.12. ANOVA Analysis

The statistical analysis was performed using the analysis of variance produced with the software Statistica10 (StatSoft, Tulsa, OK, USA). Tukey’s test was applied to detect significant differences; *p* < 0.05 was considered statistically significant.

## 3. Results and Discussion

### 3.1. Microscopic Analysis

**Figure 1:**
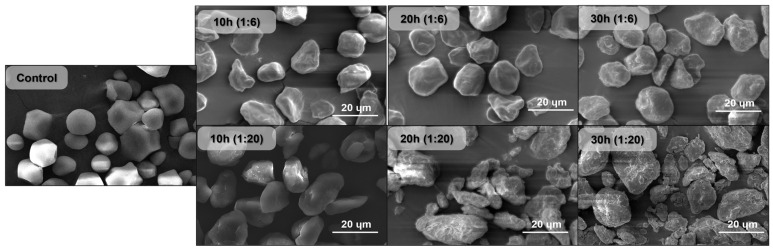
Scanning electron micrographs of starch, both control and milled samples. Scale bars: 20 μm.

The microscopic images obtained via SEM showed that the starch granule was fractured throughout the ball-milling process (Figure 1). The effects of the ball-milling process are visible in the images as the milling time increases due to the breakdown of the granules through impact, friction, and shear forces. It is important to note that there is a slight increase in the system’s temperature as the milling proceeds (37 °C at the end of the process). This increase must not have had a significant impact on the process of breaking the granules. The starch granules were oval shaped with a smooth surface. However, as the milling time increases, the surface becomes rougher, and the granule is broken down, as evidenced by the appearance of fractures of varying sizes and shapes [2].

The most fractured and heterogeneous group of granules was shown by the starch milled using the 1:20 starch/ball ratio. However, the particle size does not always decrease with milling time due to the agglomeration of the damaged starch granules. This agglomeration can be undone by suspending the starch in some solvent if the application of the material calls for it [1,3,14]. This fractured structure of the ball-milled starch gives it a larger surface area. Consequently, its reactivity increases since it is now easier for water and other chemicals to access the middle of the granule through the fractures formed during the milling process [7,12].

**Figure 2:**
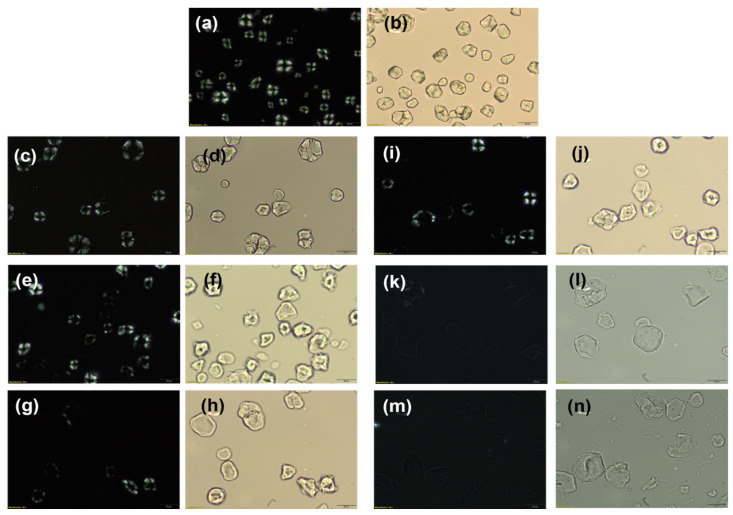
Polarized light microscope images of starch. (**a**,**b**) control; (**c**–**n**) milled starch: (**c**,**d**) 10 h (milling time), 1:6 (mass ratio of starch to balls); (**e**,**f**) 20 h, 1:6; (**g**,**h**) 30 h, 1:6; (**i**,**j**) 10 h, 1:20; (**k**,**l**) 20 h, 1:20; (**m**,**n**) 30 h, 1:20. The darker images for each pair were taken under polarized light, and the lighter images were not.

The light microscopy analysis of the control starch clearly showed birefringence (Figure 2), which is correlated to the crystalline structure of the starch granule [21]. However, as the milling time increases, the birefringence starts to fade, with complete fading in the conditions using 1:20 ratios. The complete disappearance of the birefringence indicates that the crystalline structure of the starch granules was fractured entirely, leaving a less organized amorphous structure in its place. The milling process not only superficially fractured the granule but also broke down the starch crystalline region, and this outcome might be improved further by adopting a more powerful ball mill capable of running at a higher rpm, reducing the milling time involved in the process [12,22,23].

### 3.2. Fourier Transform Infrared Spectroscopy (FTIR)

The distinctive starch bands visible in all the observed infrared spectra include the O–H stretch band at 3500–3000 cm^−1^, the C–H stretch band at 2910 cm^−1^, and the anhydroglucose stretching vibration band at approximately 950 cm^−1^, all characteristic of starch. No uncharacteristic band appeared, which indicates that the energy involved in the milling process did not cause the addition or removal of any chemical groups [15,24,25]. The ratio between the intensity of 995/1044 cm^−1^ bands was used as an indicator of the ordered structure of the starch [26]. These ratios are shown in Table 1.

**Figure 3:**
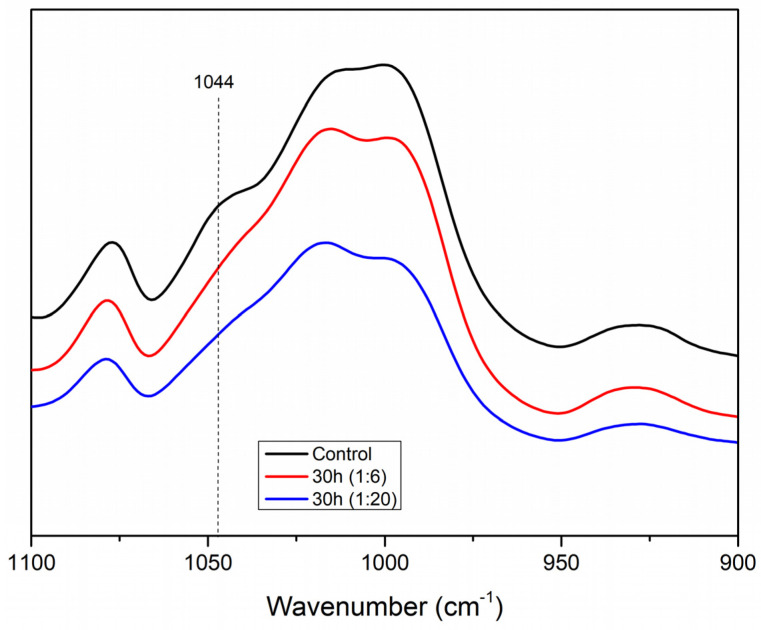
Infrared spectra in the 900 to 1110 cm^−1^ region for the samples of starch milled for 30 h (both ratios) and control starch.

Changes within this range can be utilized as a disturbance sign of the ordered structure of the starch granule because the spectral area between 1050 and 950 cm^−1^ is sensitive to the short-range double helix structural alteration [27]. Since the intensity of the 1044 cm^−1^ band diminishes as the amorphous stage increases, the 995/1044 cm^−1^ ratio grew as milling time increased by a total of 9%, indicating a drop in the starch structure order [16,28]. The most milled granules (20 h and 30 h for both ratios) also showed the highest 995/1044 cm^−1^ ratio, indicating a more amorphous structure than the other samples, corroborating the light microscopy analysis (Figure 3) and the SEM images (Figure 2). Figure 3 shows the apparent drop in the intensity of the 1044 cm^−1^ band of the 30 h milled starch (both ratios) in contrast to the control starch, which indicates the increase in the 995/1044 cm^−1^ ratio and the resulting decrease in the structural order of the starch granules.

### 3.3. Amylose Content

**Figure 4:**
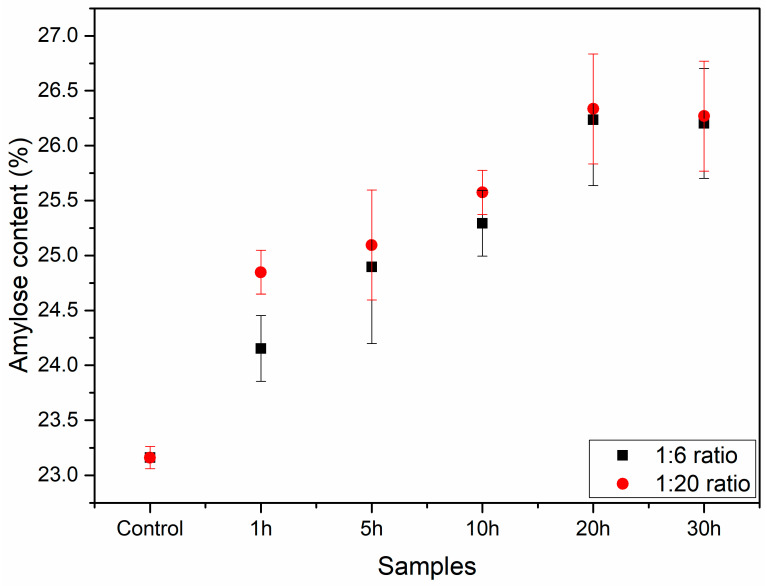
Amylose content (%) for all the starch samples, milled and control.

The amylose content peaked at 20 h of milling for both ratios and then reached a plateau (Figure 4). An increase in milling duration caused this rise in amylose content. Amylopectin, a more rigid and ramified structure, can be partially fractured in the milling process, increasing the percentage of amylose content. This rise in the amylose content may be connected to the fracture of the starch granules caused by the ball mill, as observed in the SEM analysis (Figure 2) [5,8]. The plateau reached at 20 h may be the result of the high degree of heterogeneity in the starch granule size after milling, which led to the production of numerous granules with varying amylose contents because of different degrees of breakage in each granule, as seen in the SEM images.

### 3.4. Thermogravimetric Analysis (TGA)

**Figure 5:**
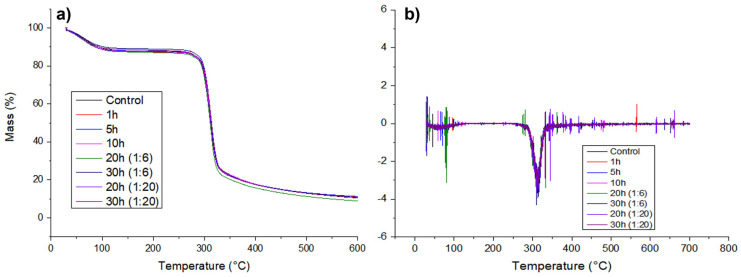
TG (**a**) and DTG (**b**) curves for control and milled starch.

Figure 5 shows all the thermograms of the studied starch samples. The first weight loss event was seen in the TG graphs at approximately 100 °C due to starch hygroscopicity, which is connected to moisture loss from the leftover water associated with the starch molecules. Next, starch degradation was observed at 300 °C, with a Tonset for all samples at approximately 297 °C, showing the breakdown of the anhydroglucose chains, which is consistent with the results for starch in the literature [29,30,31,32]. The ball-milling procedure altered the structure of corn starch, but the thermal profile remained constant at all milling times (1, 5, 10, 20, and 30 h) and ratios (1:6 and 1:20). Therefore, the thermal stability of starch was unaffected by the ball-milling process.

### 3.5. Differential Scanning Calorimetry (DSC)

Figure S1 shows the endothermic event that is clearly detected in the control sample and becomes more subtle in the samples as milling time and ratios increase. Starch gelatinization is represented by this endothermic event at approximately 70 °C. As previously stated, ball milling disturbs the crystalline structure of the starch, which can facilitate the gelatinization process, as evidenced by the DSC results [25]. As demonstrated, amylopectin degradation occurs in ball-milled starches, promoting the formation of disordered amorphous regions that are easily accessible to water and allowing gelatinization to occur at lower temperatures [33,34]. 

As milling time and ratio rise, there is a more visible change in enthalpy, not only does the gelatinization process begin at a lower temperature but less energy is required to gelatinize the milled starch (Table 2). The peak is essentially nonexistent in the samples milled at a 1:20 ratio for 10, 20, and 30 h showing that the sample gelatinized, at least partially, during the period it was resting in water prior to measurement and thus gelatinized at ambient temperature. These results corroborated with the FTIR and microscopy results, suggest a partial disturbance in the starches’ crystalline structure which makes the gelatinization process easier, but this crystalline disturbance is not accentuated enough for it to have a direct effect on overall thermal stability and the degradation of the anhydroglucose chains, evidenced by the similar thermal profile obtained in the TGA results (Figure 5) [35,36]. 

### 3.6. X-ray Diffraction (XRD)

**Figure 6:**
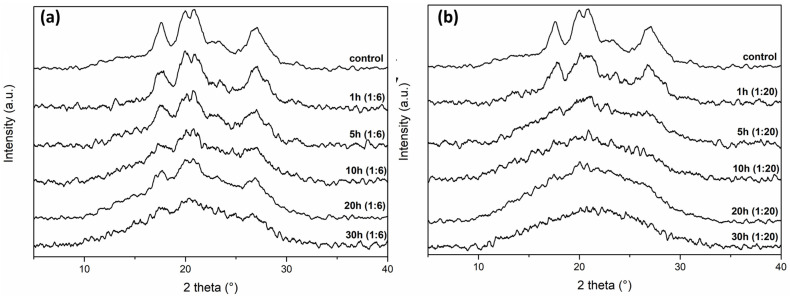
Diffractograms of the 1:6 (**a**) and 1:20 ratio (**b**) milled starch samples and the control sample.

The diffractograms of the examined samples are shown in Figure 6, which reveals the expected A-type crystal structures typical of corn starch. A doublet around 19–20° and significant crystalline peaks at about 17° and 27° can be seen in the diffractograms. Minor variations in peak degrees are seen, which are probably caused by the analysis using Co tubes rather than Cu tubes [37,38].

For both ball/mass ratios, the crystalline peaks showed reduced intensity as milling time increased, indicating a decline in structural organization and resulting in a lower crystalline index (CrI), as shown in Table 3. For shorter milling periods, the higher ball/mass ratio had a more significant impact on the CrI, as shown by a visible disturbance in the peaks and, consequently, lower CrI values [16]. The decrease in CrI supports the polarized microscopy results (Figure 2), which demonstrate that the milling procedure successfully decreased the crystallinity of the starch samples.

### 3.7. Swelling Power (SP) and Solubility (S%)

**Figure 7:**
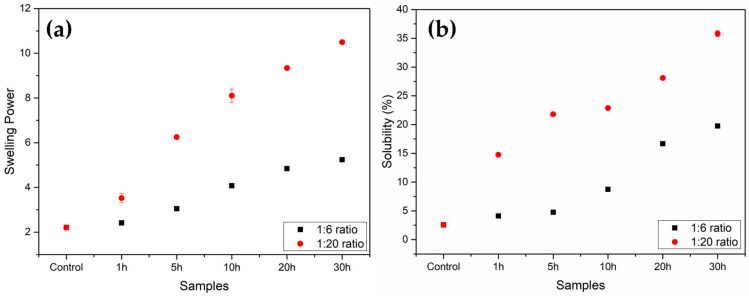
Swelling power (**a**) and solubility (**b**) of the native corn starch sample (Control) and the ball-milled starch samples.

Figure 7 shows that as the ball milling time and ratio rose, the swelling power (SP) and solubility (S%) of the samples increased. The ball-milling procedure gradually enhanced the capacity of the starch granules to retain water in their structure. A significant difference is evident between the 1:6 ratio and 1:20 ratio samples due to the increased fragmentation of the structures, as observed by the SEM images (Figure 1).

The starch crystalline structure is broken when it is heated in excess water due to the breakdown of intramolecular and intermolecular hydrogen bonds. Water molecules then establish hydrogen bonds with the exposed hydroxyl groups of amylose and amylopectin, causing granule swelling to rise. The amount of interaction between starch chains within the amorphous and crystalline domains has a major impact on swelling power [20,39]. Moreover, the complete or partial breakage of the starch crystalline structure via ball milling proved to have a similar effect to temperature on the SP and S% results. The granules become more fragmented as both the time and ratio of ball milling increase (Figure 1), making the structure more accessible to water. This can be correlated with the partial destruction of the amylopectin chains and changes in the amylose content since both properties are associated with the swelling power capacity and solubility of the starch. The water, even at room temperature, can easily permeate the granule (in the more-milled samples), causing the swelling power and solubility to rise significantly from the native starch (control) to, especially, the 1:20 ratio samples. This shows SP and S% values compatible with those of gelatinized starch mentioned in other studies [19,20,39,40].

### 3.8. Rheology

Table 4 lists all the rheological data acquired for the starch samples, both control and milled at various times and ratios. The gelatinization temperature of the starch (TGel) decreased continuously as both time and ratio increased, resulting in a pregelatinized starch generated purely through milling. The gelatinization of the 20 and 30 h samples (1:20 ratio) was not captured by the equipment because the samples were gelatinized simply by mixing with water as a preparation for the analysis at room temperature (25 °C), as deducted from the DSC analysis (topic 3.5).

During temperature and frequency sweep testing of starch suspensions, the dynamic rheometer enables continuous assessment of dynamic moduli and viscosity. The storage modulus (G′) is a measure of the energy stored and recovered from the material every cycle, whereas the loss modulus (G″) is a measure of the energy dissipated or lost per cycle of sinusoidal deformation [41]. When compared to the control sample, the viscosity, G′, and G″ increased at the beginning of the milling process (1, 5, and 10 h ratio 1:6), indicating that partial fragmentation of the starch via ball milling facilitates the gelatinization process, forming a more viscous gel after sample preparation (90 °C for 10 min). However, as milling time increases, the viscosity, G′ and G″ begin to decrease, indicating that while initial fragmentation causes the starch to form a more viscous gel, as the starch is further fragmented, this gel begins to lose viscosity, reaching a minimum in the 30 h 1:20 ratio sample. This drop in viscosity, as well as G′ and G″, is because the amylopectin chain decreases as the granule is broken, so the gel generated by this starch is less viscous than those made by less-milled samples [22,30,42].

Thixotropy is a property associated with starch gel and paste rheoinstability. Simply put, thixotropy measures how well or poorly a gel or paste may return to its initial conformation after being subjected to a shear rate fluctuation. The greater the thixotropy, the greater the gap between the initial and end states and the higher the rheoinstability of the gel or paste. The thixotropy of the samples decreased as the milling time and ratio increased, reaching low numbers (<2.2) for the 10, 20, and 30 h (1:20 ratio) samples. This behavior occurs because the more-milled samples have smaller granules, and their structure is formed by weaker bonds that can be easily restored once the sheer rate decreases, whereas the less-milled and control samples have larger granules and thus more complex bonds that would take more time to completely restore, if ever [35,43,44]. All the rheology curves for all the samples can be found in the Supplementary Material (Figures S2–S4).

### 3.9. Film Forming Ability

**Figure 8:**
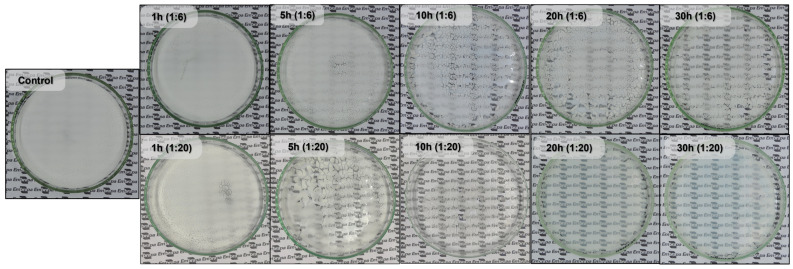
Films formed by oven drying starch suspensions (10%) at room temperature (30 °C) using control and milled starches.

Figure 8 shows films formed by all starch samples and evidence of how the milling time affected the starch ability to gelatinize at room temperature (30 °C) and create films. The control starch suspension, once dried, forms a thin opaque layer of starch powder. However, as the milling time increased, the starch suspensions dried and formed more uniform and transparent films. The 10, 20, and 30 h milled samples at a 1:20 ratio produced the best-looking films, but the 20 h (1:20 ratio) is the one that showed a completely homogenous and see-through film with the shortest milling time possible, with 10 h (1:20 ratio) being a close second, since its film has some parts where the starch was not completely gelatinized. To the best of the authors’ knowledge, no work has described, in depth, the impact of milling on the gelatinization of starch, despite the impressive discoveries reported in the literature about starch milling.

According to Goiana et al. and Oliveira et al. [15,25], most studies that use starch films heat the starch and water to approximately 90 °C for starch gelatinization before adding a plasticizing agent, such as glycerol, to ensure film resilience. However, milled starches with a lower gelatinization temperature were able to form a thin and fragile film without heating or the addition of any plasticizing agents.

A more fractured granule has a larger surface area and is more susceptible to the effects of water. Water can enter the grain quickly, swelling it and causing gelatinization of the starch. This behavior displayed by the milled starch is related to the fracture caused by the milling process. Therefore, milling is an efficient way to reduce the starch gelatinization temperature [9,10,11].

## 4. Conclusions

Ball milling altered the starch granule physical makeup by shattering it. These fractures made it easier for water to access the granule, boosting the granule’s reactivity and decreasing its gelatinization temperature. The 10 h milled starch at a 1:20 starch/ball mass ratio underwent enough physical and gelatinization temperature changes, with the shortest milling time possible, to be used as a thickening agent in thermally sensitive applications, such as the pharmaceutical industry and some food products. However, samples milled at different times can have different applications, such as the 1 h milled starch at a 1:6 mass ratio forming a more viscous gel than the control starch, making it a more efficient thickening agent than the non-milled starch. In addition, this research made a discovery: a process as simple as milling starch can drastically change the gelatinization temperature of starch leading to effects on its rheological properties.

## Figures and Tables

**Table 1:** The 995/1044 cm^−1^ band ratio for control and milled starch.

Sample	995/1044 cm^−1^ (%)	Sample	995/1044 cm^−1^ (%)
Control	64.9 ± 0.1 ^d^	1 h (1:20 ratio)	65.6 ± 0.6 ^c,d^
1 h (1:6 ratio)	65.4 ± 0.5 ^c,d^	5 h (1:20 ratio)	66.9 ± 0.3 ^c^
5 h (1:6 ratio)	66.4 ± 0.6 ^c^	10 h (1:20 ratio)	67.9 ± 0.5 ^c^
10 h (1:6 ratio)	66.9 ± 0.2 ^c^	20 h (1:20 ratio)	70.0 ± 0.9 ^b^
20 h (1:6 ratio)	69.5 ± 0.3 ^b^	30 h (1:20 ratio)	73.9 ± 1.2 ^a^
30 h (1:6 ratio)	68.9 ± 0.7 ^b^	--------	--------

Values not marked by the same letter are significantly different (*p* < 0.05).

**Table 2:** Gelatinization enthalpy, peak, and end temperature of starch samples.

Sample	ΔHgel (J·g^−1^)	Peak Temperature (°C)	End Temperature (°C)
Control	1.86 ± 0.21 ^a^	71.4	83.5
1 h (1:6 ratio)	1.75 ± 0.15 ^a^	69.6	82.4
5 h (1:6 ratio)	1.48 ± 0.11 ^b^	69.2	79.4
10 h (1:6 ratio)	0.76 ± 0.10 ^d^	64.9	77.7
20 h (1:6 ratio)	0.72 ± 0.09 ^d,e^	65.7	77.6
30 h (1:6 ratio)	0.64 ± 0.07 ^e^	61.8	74.6
1 h (1:20 ratio)	0.88 ± 0.05 ^c^	69.9	81.2
5 h (1:20 ratio)	0.84 ± 0.09 ^c^	65.3	77.3
10 h (1:20 ratio)	0.38 ± 0.06 ^f^	62.3	75.1
20 h (1:20 ratio)	0.24 ± 0.08 ^f^	61.9	74.3
30 h (1:20 ratio)	0.01 ± 0.00 ^g^	61.5	66.7

Values not marked by the same letter are significantly different (*p* < 0.05).

**Table 3:** Crystalline indexes (CrI) of the control and milled starch samples.

Sample	CrI (%)	Sample	CrI (%)
Control	34.8	1 h (1:20 ratio)	28.6
1 h (1:6 ratio)	31.9	5 h (1:20 ratio)	12.9
5 h (1:6 ratio)	27.0	10 h (1:20 ratio)	8.3
10 h (1:6 ratio)	15.9	20 h (1:20 ratio)	2.8
20 h (1:6 ratio)	10.7	30 h (1:20 ratio)	2.7
30 h (1:6 ratio)	8.4	--------	--------

**Table 4:** Rheological parameters of the control and milled starch samples.

Samples	TGel (°C)	Viscosity (ɳ) [cP]	Storage Module (G′) [Pa]	Loss Module (G″) [Pa]	Thixotropy
Control	69.1	889.7	2.24	5.12	27.02
1 h (1:6 ratio)	63.4	1601.00	3.33	9.49	23.77
5 h (1:6 ratio)	61.3	921.20	1.32	5.64	17.38
10 h (1:6 ratio)	55.9	969.90	1.28	5.96	9.89
20 h (1:6 ratio)	51.6	255.90	0.28	1.58	9.15
30 h (1:6 ratio)	50.1	119.20	0.12	0.74	4.75
1 h (1:20 ratio)	62.7	660.70	1.24	3.97	16.74
5 h (1:20 ratio)	60.1	352.90	0.76	2.09	11.14
10 h (1:20 ratio)	51.4	173.20	0.35	1.03	2.04
20 h (1:20 ratio)	*	148.60	0.23	0.91	2.16
30 h (1:20 ratio)	*	81.62	0.15	0.49	−0.07

* Samples gelatinized at room temperature during sample preparation.

## Data Availability

The data used to support the findings of this study can be made available by the corresponding author upon request.

## Supplementary Materials

The following supporting information can be downloaded at: https://www.mdpi.com/article/10.3390/foods12152924/s1; Figure S1: Differential scanning calorimetry curves for control and milled starch; Figure S2: Gelatinization temperature rotational rheology experiment for all the starch samples; Figure S3: Viscosity, storage modulus (G′), and loss modulus (G″) oscillatory rheology experiment for all the starch samples; Figure S4: Shear rate vs. stress rheology curves for all the starch samples, for thixotropy determination.
