# Supplementary material for: Effect of Ball-Milling on Starch Crystalline Structure, Gelatinization Temperature, and Rheological Properties: Towards Enhanced Utilization in Thermosensitive Systems
Source: Foods. 2023 Jul 31;12(15):2924. doi: 10.3390/foods12152924 (PMC10417754; doi:10.3390/foods12152924)
Supplement: Supplementary file 1 [file foods-12-02924-s001.zip › foods-2480596-supplementary.pdf]

SUPPLEMENTARY MATERIAL

Effect of Ball-Milling on Starch Crystalline Structure, Gelatinization Temperature, and Rheological Properties: Towards Enhanced Utilization in Thermosensitive Systems

Matheus de Oliveira Barros [1], Adriano Lincoln Albuquerque Mattos [2], Jessica Silva de Almeida [1], Morsyleide de Freitas Rosa [2] and Edy Sousa de Brito [2,3,*]

[1] Department of Chemical Engineering, Federal University of Ceará (UFC), Fortaleza CEP 60455-760, Brazil; matheus.oliveira_@hotmail.com.br (M.d.O.B.); jeh.quimica@gmail.com (J.S.d.A.)
[2] Embrapa Tropical Agroindustry, Rua Dra Sara Mesquita 2270, Fortaleza CEP 60511-110, Brazil; adriano.mattos@embrapa.br (A.L.A.M.); morsyleide.rosa@embrapa.br (M.d.F.R.)
[3] Embrapa Food and Territories, Rua Cincinato Pinto 348, Maceió CEP 57020-050, Brazil
* Correspondence: edy.brito@embrapa.br

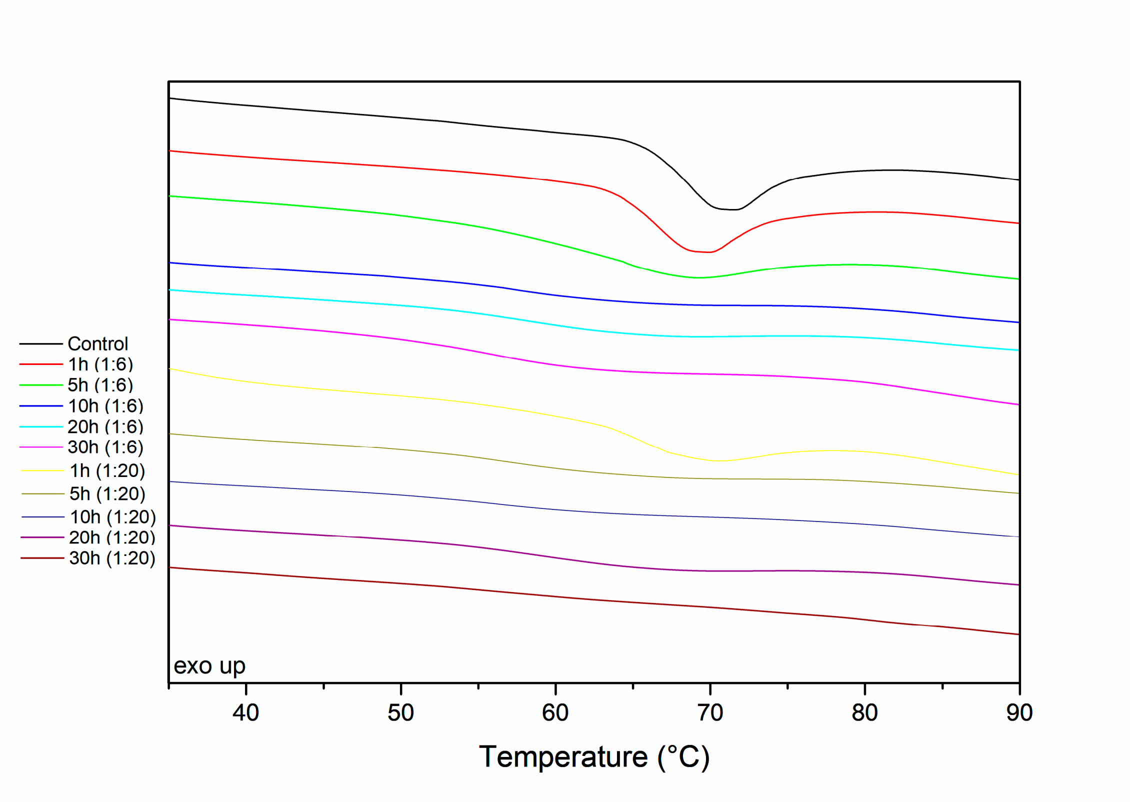

Figure S1. Differential scanning calorimetry curves for control and milled starch.

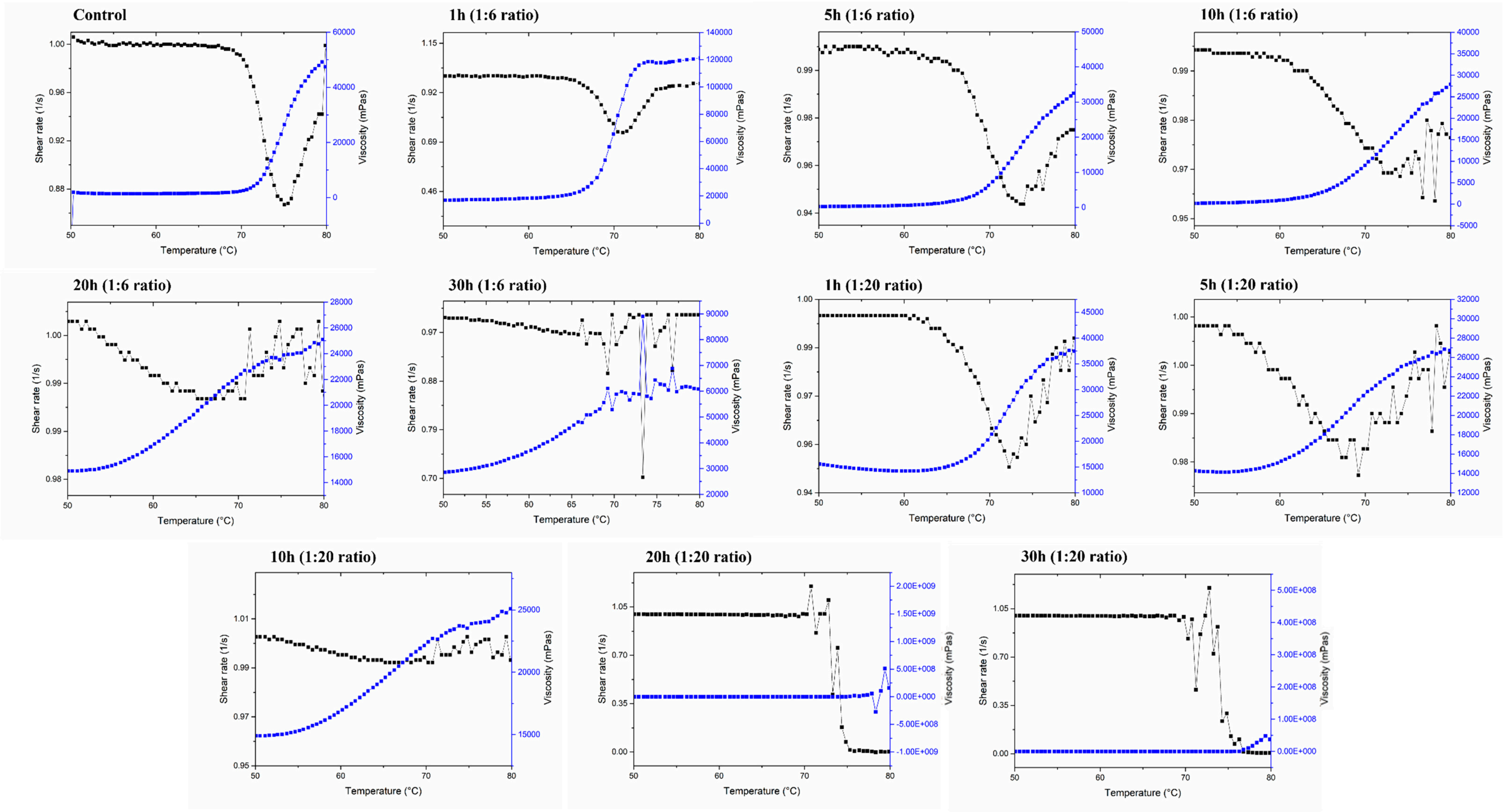

Figure S2. Gelatinization temperature rotational rheology experiment for all the starch samples.

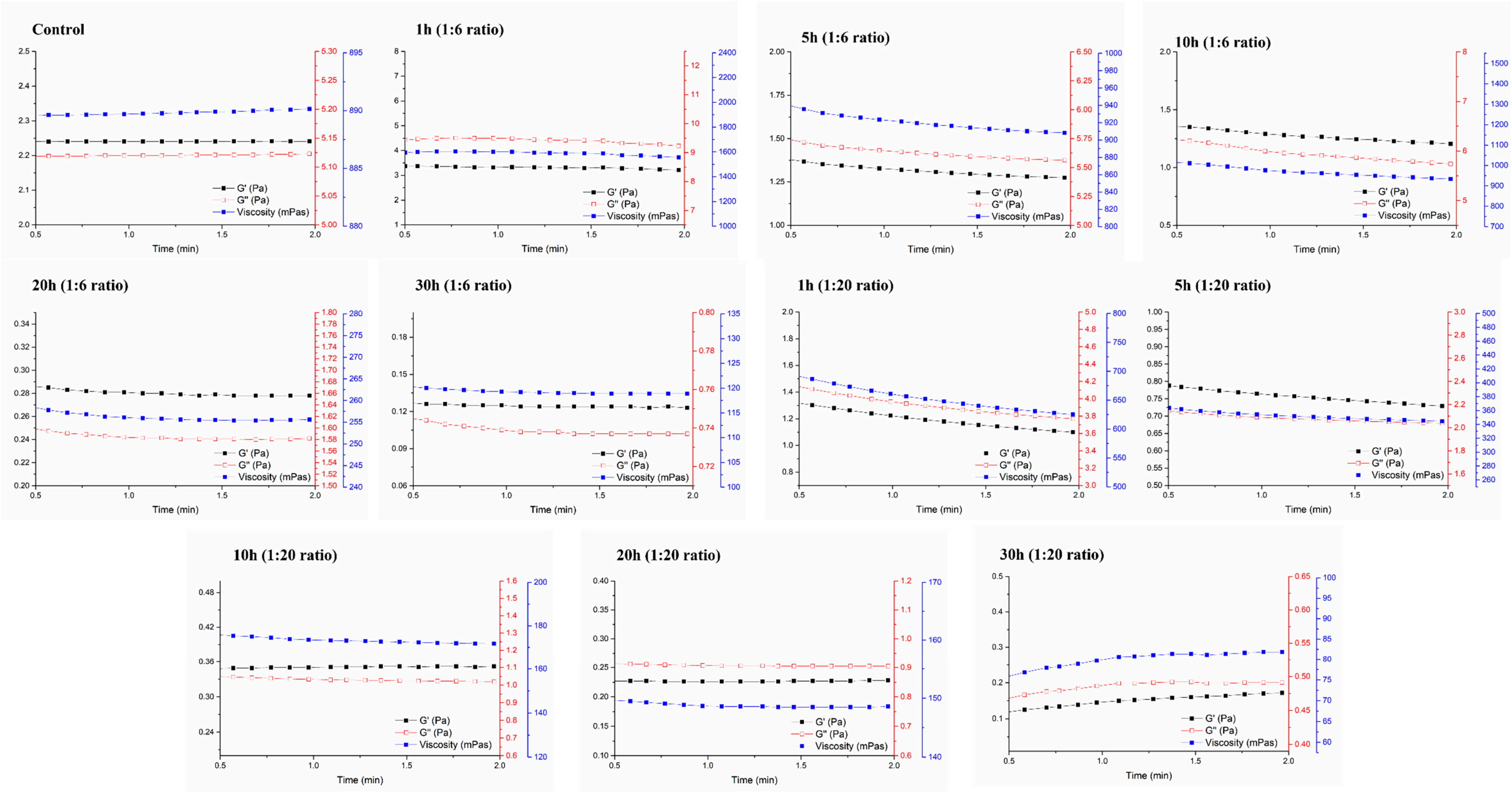

Figure S3. Viscosity, storage modulus (G'), and loss modulus (G'') oscillatory rheology experiment for all the starch samples.

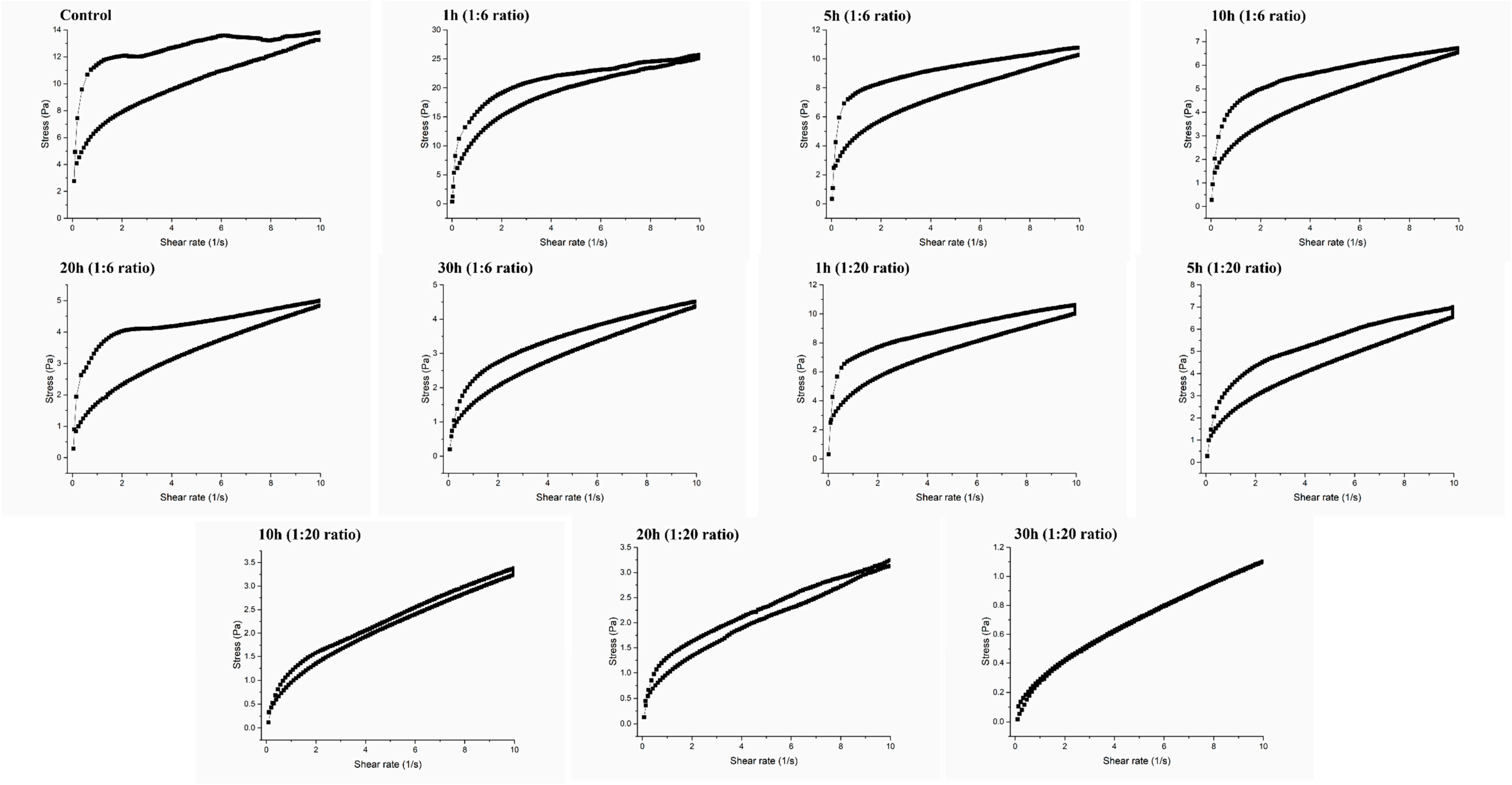

Figure S4. Shear rate vs. stress rheology curves for all the starch samples, for thixotropy determination.
